# Involvement of *MoVMA11*, a Putative Vacuolar ATPase c’ Subunit, in Vacuolar Acidification and Infection-Related Morphogenesis of *Magnaporthe oryzae*

**DOI:** 10.1371/journal.pone.0067804

**Published:** 2013-06-27

**Authors:** Guoqing Chen, Xiaohong Liu, Lilin Zhang, Huijuan Cao, Jianping Lu, Fucheng Lin

**Affiliations:** 1 State Key Laboratory for Rice Biology, Biotechnology Institute, Zhejiang University, Hangzhou, China; 2 College of Life Sciences, Zhejiang University, Hangzhou, China; 3 China Tobacco Gene Research Center, Zhengzhou Tobacco Institute of CNTC, Zhengzhou, China; Soonchunhyang University, Republic of Korea

## Abstract

Many functions of vacuole depend on the activity of vacuolar ATPase which is essential to maintain an acidic lumen and create the driving forces for massive fluxes of ions and metabolites through vacuolar membrane. In filamentous fungus *Magnaporthe* *oryzae* , subcellular colocalization and quinacrine staining suggested that the V_1_V_0_ domains of V-ATPase were fully assembled and the vacuoles were kept acidic during infection-related developments. Targeted gene disruption of *MoVMA11* gene, encoding the putative c’ subunit of V-ATPase, impaired vacuolar acidification and mimicked the phenotypes of yeast V-ATPase mutants in the poor colony morphology, abolished asexual and sexual reproductions, selective carbon source utilization, and increased calcium and heavy metals sensitivities, however, not in the typical pH conditional lethality. Strikingly, aerial hyphae of the *MoVMA11* null mutant intertwined with each other to form extremely thick filamentous structures. The results also implicated that *MoVMA11* was involved in cell wall integrity and appressorium formation. Abundant non-melanized swollen structures and rare, small appressoria without penetration ability were produced at the hyphal tips of the *ΔMovma11* mutant on onion epidermal cells. Finally, the *MoVMA11* null mutant lost pathogenicity on both intact and wounded host leaves. Overall, our data indicated that *MoVMA11*, like other fungal *VMA* genes, is associated with numerous cellular functions and highlighted that V-ATPase is essential for infection-related morphogenesis and pathogenesis in *M* *. oryzae* .

## Introduction

Vacuolar H^+^-ATPases (V-ATPases) are multisubunit enzymes composed of a peripheral ATPase sector (V_1_) and a membrane-bound proton-translocating sector (V_0_) [1,2]. In response to glucose deprivation, V_1_ V_0_ sectors can reversibly dissociate via cytosolic pH transferring the starved signal to V-ATPase complexes [3,4]. Yeast V_1_ sector includes eight different subunits, designated A-H, whereas the V_0_ sector is comprised of subunit a, d, e, and the proteolipid c ring, which contains subunit c (vma3), c’ (vma11), and c″ (vma16). In fungi, proteolipid c/c’ and c″ subunits descend from two gene duplications of a common ancestor gene [5], and make up a hexameric ring in a ratio of 4 (c): 1 (c’): 1 (c″) with a specific orientation [6]. But in higher eukaryotic cells, subunit c’ is absent from the proteolipid ring, which is composed of five c copies and a single c″ subunit instead [5,7]. With the aid of dedicated assembly factors, yeast V_0_ sector is assembled in endoplasmic reticulum (ER) independently (or coordinately with V_1_ subunits) prior to trafficking to Golgi apparatus for the full assembly of V-ATPase holoenzymes [7,8]. Apart from Golgi-derived secretory vesicles, eukaryotic V-ATPases also reside and function on other intracellular compartments, including lysosomes/lysosome-like vacuoles, early and late endosomes [8,9].

V-ATPase-driven pH homeostasis of intracellular compartments is crucial for massive transmembrane transport of ions and metabolites, vesicular trafficking, and many other cellular processes [7–9]. Disruption of V-ATPase function in *Saccharomyces cerevisiae* leads to a characteristic pH-dependent phenotype, the Vma^-^ phenotype [10,11]. Yeast *vma* mutants do not grow at alkaline pH and/or in high concentrations of extracellular calcium, or on non-fermentable carbon sources, and are acutely sensitive to a variety of heavy metals. Other fungi, such as *Schizosaccharomyces pombe*, *Candida albicans*, and *Neurospora crassa*, also show numbers of growth defects upon loss of V-ATPase activity [12–14]. Except for certain tissue-specific isoforms, systemic V-ATPase genes are even critical for the survival of higher eukaryotes [15]. Although V-ATPase complexes have been identified throughout eukaryotes [16], however, comparatively little has been done on possible relationships between V-ATPase and fungal plant infection.

Rice blast, caused by *Magnaporthe* *oryzae* , is one of the most serious rice diseases that cause substantial cultured crop losses worldwide. Genomic sequence availability and genetic tractability of both *M* *. oryzae* and rice, combined with multiple analytical tools, make them a model plant pathosystem for fungus–plant interaction research [17–19]. Multiple yeast anatomized signal transduction pathways have been identified that are highly conserved and found to also control the infection-related morphogenesis in *M* *. oryzae* [20,21]. Among them, cAMP/protein kinase A (PKA) signaling pathway is involved in not only asexual and sexual reproduction, but also host surface recognition and rapid mobilisation of lipid and glycogen storages during appressorium formation [22–24]. Meanwhile, fungal vacuoles are long-recognised critical for cellular homeostasis, membrane trafficking and protein turnover [25,26]. Appressorium of *M* *. oryzae* , formed at germ tube tip of three-celled conidium, can generate a turgor pressure as high as 8 MPa through vacuolar degradation of stored lipid reserves [27]. Differentiation of functional appressorium requires autophagic cell death of the conidium, and vacuoles act as a sink for autophagosomes degradation [28–30]. As described above, all of the features of fungal vacuoles are closely related to V-ATPase activities. Besides, V-ATPase is recently identified as a novel upstream regulator of PKA pathway in both yeast and certain mammalian cells [4].

In this study, *M* *. oryzae* V-ATPase genes were characterized and investigated by gene expression profiling and subcellular localization. *MoVMA11*, putatively encoding the subunit c’ of V-ATPase, was further deleted to unveil its functions during the growth and development of *M* *. oryzae* . Our results of *MoVMA11* null mutant demonstrate that the V-ATPase complex with its role in the building and maintenance of pH gradient is essential for vacuolar detoxification, hyphal growth, conidia and ascospore production, and pathogenesis in *M* *. oryzae* .

## Materials and Methods

### Strains and culture conditions

*M* *. oryzae* wild-type (WT) strain Guy11 and all the derivative transformants were maintained on CM agar plates at 26 °C with a 16 h fluorescent light photophase [31]. Genetic crosses between *M* *. oryzae* WT-derived strains and 2539 were carried out on oatmeal medium (3% oatmeal and 0.5% glucose) [32]. Growth phenotypic comparisons of WT and *ΔMovma11* strains were performed on MM supplemented with various ions (200 mM Ca2^+^, 1 mM Cu^2+^, 3 mM Fe^2+^, 3 mM Mn^2+^, and 4 mM Zn^2+^) and a series of glucose-substituted carbon sources, or CM containing cell wall perturbing agents (200 μg/ml Calcofluor white, 200 μg/ml Congo red, and 0.01% SDS). To test the pH sensitivity, strains were grown on MM or CM buffered to pH 5.6-8.2 using 20 mM HEPES [14]. Genomic DNA was extracted from mycelia cultured in liquid CM for 3-4 days.

### Quantitative RT (qRT)-PCR assay

Fungal tissues used for qRT-PCR analysis included vegetative mycelia harvested from 3-day-old cultures in liquid CM, conidia collected from 10-day-old CM plate cultures, appressoria formed on hydrophobic surfaces 24 hours postincubation (hpi), and infected barley leaves harvested 3-4 dpi. Total RNAs of the above samples were isolated with the Trizol reagent (Takara) following a previously described protocol [33]. After the synthesis of first strand cDNA from 800 ng of total RNA using SYBR ExScriptTM RT-PCR kit (Takara), real-time PCR reaction was performed with SYBR Premix Ex Taq (Takara) on a Mastercycler ep realplex thermo cycler (Eppendorf) [34]. Relative abundance of transcripts was calculated by the 2^-ΔΔCt^ method [35] with β-tubulin (MGG_00604) as the endogenous control. Data were collected from at least two independent experiments with four replicates, and a representative set of results was presented. Primer pairs used for qRT-PCR analysis are listed in Table S1.

### Generation of *MoVMA11* gene deletion vector and mutants

The *MoVMA11* gene deletion vector was constructed following a strategy based on double-joint PCR [36]. Primers VMA11up-1/2 and VMA11dn-1/2 were used to amplify the 1.1 kb upstream and 1.1 kb downstream flanking sequences of the *MoVMA11* locus from genomic DNA, respectively. A 1.4 kb *hph* cassette was cloned from pCB1003 with primers HPH-1/2. The three amplicons were joined together in the second round of PCR, the product of which served as the template for the final construct amplification with nested primers nVMA11-1/2. The double-joint PCR product was inserted into the PstI/SalI sites of pCAMBIA1300 to obtain the targeted gene deletion vector, which was introduced into *M* *. oryzae* WT strain via *Agrobacterium tumefaciens*-mediated transformation (ATMT) [37]. After PCR screening, putative *Movma11* null mutants were further confirmed by Southern blot analysis. For complementation of the deletion strain, a fragment containing genomic sequences of the *MoVMA11* locus along with its promoter and terminator regions was amplified with primers VMA11-C1/2, and inserted into a modified pCAMBIA1300 vector, which contained a geneticin resistance gene. The resulting construct was randomly inserted into the genome of the *ΔMovma11* mutant using the ATMT method. Southern blot analysis was carried out to verify successful single-copy integration according to the manufacturer’s instructions of the digoxigenin (DIG) high prime DNA labeling and detection starter kit I (Roche).

### Construction of Movma11, Movma16, and Movma2-RFP fusion plasmids

For a better visualization of the intracellular distribution pattern of the target protein-GFP/RFP, we expressed the fused proteins under the control of the histone H3 (MGG_01159.7) promoter. The *H3* promoter region was amplified from the *Magnaporthe* genomic DNA with primers H3-1/2, and inserted into the EcoRI/SalI sites of pCAMBIA1300 to produce a plasmid, pKD. To generate the GFP expression vector, eGFP was amplified with primers eGFP-1/2 from pEGFP (clontech), and a 2.8 kb fragment containing a sulfonylurea resistance allele of *Magnaporthe* *ILV1* gene was amplified with SUR-1/2 from pCB1528; subsequently, the fragments were inserted into the SmaI/XbaI or XhoI/EcoRI sites of pKD, respectively, to obtain the recombinant vector pKD5. pKD6, a RFP expression plasmid conferring geneticin resistance, was constructed using the same strategy with primers DsRED-1/2 and NEO-1/2. Coding sequences of *MoVMA11* and *MoVMA16* were amplified with VMA11N-1/2 and VMA16N-1/2, and cloned into the BamHI/SmaI sites of pKD5 to generate the GFP C-terminal tagged fusion construct pKD51 and pKD52, respectively. Similarly, primers VMA2N-1/2 were used to amplify the *MoVMA2* cDNA, which was inserted into the SmaI site of pKD6 to obtain pKD61. Vector pKD52 was not only transformed separately, but also co-transformed with pKD61 into WT, while pKD51 was introduced into the *Movma11* null mutant (or with pKD61). Transformants were verified by GFP expression screening and Southern blot analysis.

### Staining methods and microscopy

Appropriately diluted conidia (~1×10^5^/ml), collected from CM agar plate, were incubated onto hydrophobic films in a moist chamber at room temperature. To stain nuclei, samples were soaked in 1 μg/ml DAPI (2,4, -Diamidino-phenyl-indole) solutions in the dark for 5 min before epifluorescence microscopy examination. For vacuolar staining, conidia were incubated with 7.5 μM FM4–64 (*N*-(3-triethylammoniumpropyl)-4-(*p*-diethylaminophenyl-hexatrienyl) pyridinium dibromide) on hydrophobic surfaces for 2 h before the solution was gently replaced by sterile distilled water, and vacuoles were observed at different time points (e.g. 2, 6 and 24 h) [38]. Vacuolar luminal dye CMAC (7-amino-4-chloromethylcoumarin) was used as previously described [39]. Quinacrine staining method was modified from that used for yeast in previous studies [40,41]. Strains were firstly grown in liquid CM on glass slides for 24 h, and then stained with the quinacrine staining solution at room temperature for 15 min. The quinacrine staining solution was prepared by adding 200 μM quinacrine (Sigma-Aldrich) into liquid CM containing 100 mM HEPES (pH 7.6) or 100 mM MES (pH 7.7). Before microscopic examination, hyphae were washed three times with ice-cold 100 mM HEPES (pH 7.6) or 100 mM MES (pH 7.7) plus 2% glucose.

An Eclipse 80i microscope (Nikon) equipped with Plan APO VC 100X/1.40 oil objective was used for light and epifluorescence microscopic examination.

### Assays for conidiation, appressorium formation and pathogenicity

Quantitative measurement of conidial production was performed with 7-day-old cultures grown on CM plates [42], while aerial hyphal and conidial development was monitored as previously described [43].

As conidiation was abolished in the *ΔMovma11* mutant, mycelial suspension, rather than conidial suspension, was placed on plastic cover slips (Fisher) or onion epidermal cells under humid conditions at room temperature for appressorial development tests. Mycelial suspension was prepared by culturing conidia and/or fragmented aerial mycelia, harvested from fungal agar plates, in liquid CM for 2 days, and then washing the cultured mycelia twice with sterile distilled water. Appressoria, formed at hyphal tips, or appressorial penetration and invasive growth were observed and photographed with a light microscope.

For plant infection assays, mycelial agar plugs were incubated on the intact or wounded rice (*Oryza sativa* cv. CO39) or barley leaves, and lesion formation was examined at 4-5 dpi.

## Results

### Identification and expression profile of V-ATPase genes in *M* *. oryzae*

Using protein sequences of *S. cerevisiae* V-ATPase subunits for BLASTP searches, we identified the repertoire of V-ATPase encoding genes in the *M* *. oryzae* genome (http://www.broadinstitute.org/annotation/genome/magnaporthe_comparative/MultiHome.html). In general, *M* *. oryzae* V-ATPase proteins are evolutionarily conserved and the majority show at least 35% sequence identity, mostly in the conserved regions, to their yeast counterparts at the amino acid level (Table S2). In addition, these proteins possess characteristic features of V-ATPase subunits as recognized by InterPro (http://www.ebi.ac.uk/InterPro), while none of them, even subunit a, is present as multiple isoforms (Table S2).

**Figure 1:**
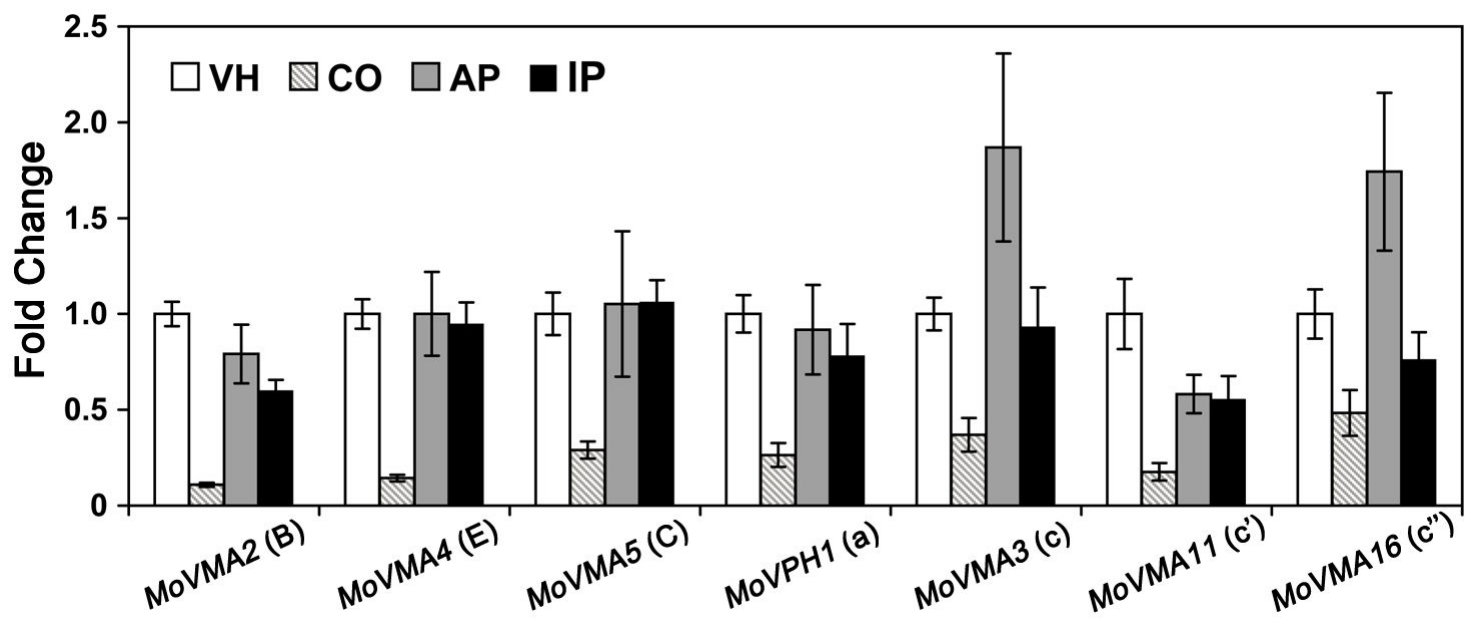
Relative transcript abundances of seven V-ATPase genes in different developmental stages. Quantitative PCR assays were carried out with RNA samples obtained from different developmental stages of WT strain, including vegetative hyphae (VH), conidia (CO), appressoria (AP), and infected plant leaves (IP). Gene expression levels were normalized by using the β-tubulin gene as an internal standard and calibrated against the VH profile for each condition. Data are representative of at least two independent experiments with similar results, and the error bars represent standard deviations of four replicates.

Gene expression patterns of several V-ATPase subunits, including subunit B, C, E, a, and the three proteolipid subunits c-c’’, were evaluated by qRT-PCR assays in vegetative hyphae, conidia, appressoria, and infected plant leaves (Figure 1). All the tested V-ATPase genes shared similar expression profiles in the four different stages of fungal development. Compared to vegetative hyphae, these genes were down-regulated by more than two-fold in conidia, but the transcriptional differences were insignificant in appressoria or infected plant leaves. V-ATPase down-regulation indicated that conidial vacuoles were not kept as acidic as those of other fungal tissues, which would prevent the vacuolar degradation of the nutrients stored in conidia.

### Subcellular location of three V-ATPase subunits in *M* *. oryzae*

**Figure 2:**
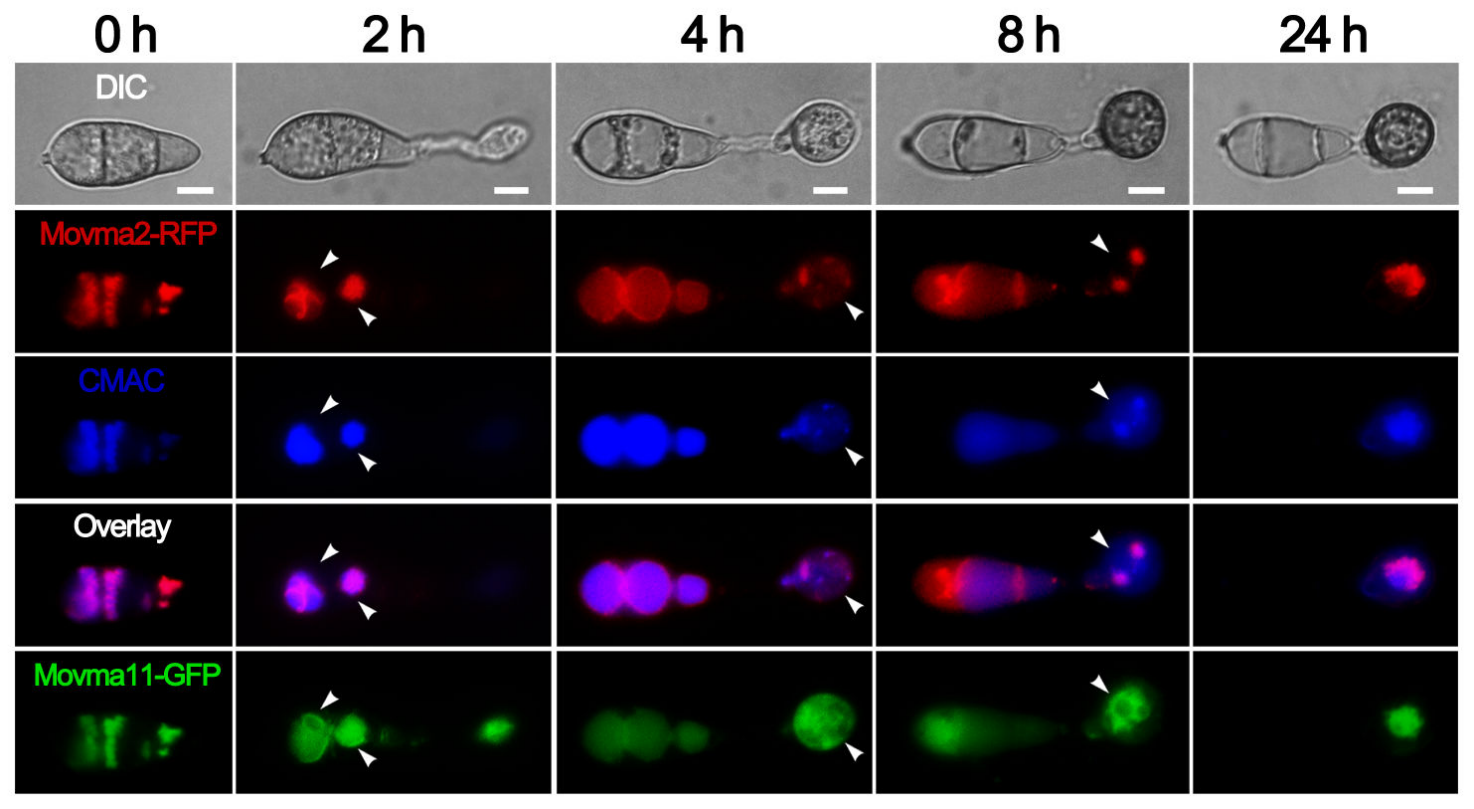
Subcellular location of Movma2 and **Movma11 proteins during appressorium development.** Conidia of *M. oryzae* strain, expressing both Movma11-GFP and Movma2-RFP, were incubated on the surfaces of hydrophobic films, and CMAC staining of vacuoles was performed at the indicated time points. The merged panels show strong colocalization of Movma2-RFP with vacuoles that are stained with CMAC. Arrowheads point to the CMAC-negative structures visualized by Movma11-GFP. Bars = 5 μm.

**Figure 3:**
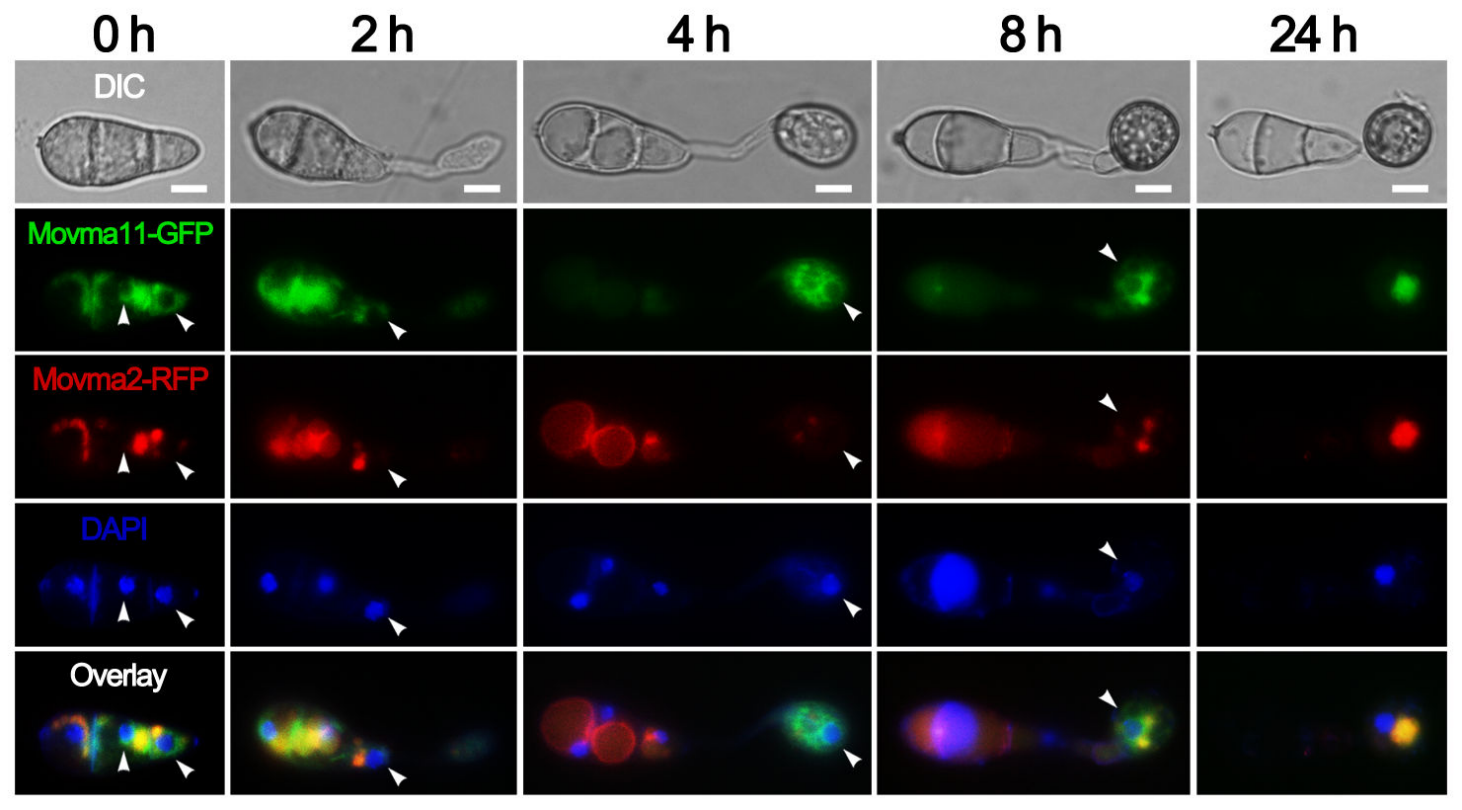
DAPI staining of strain expressing both Movma11-GFP and Movma2-RFP. DAPI was used to stain the nuclei of *M. oryzae.* Appressorial development was examined at the indicated time points. Arrowheads indicate the Movma11 resident compartments located around the nuclei that do not colocalize with Movma2. Bars = 5 μm.

To examine the distribution pattern of V-ATPase subunits, we inserted the GFP fusion cassette at C terminus of the native genomic *MoVMA11* locus by recombination strategy [44]. However, the GFP fusion strain showed a weak fluorescence. To achieve a better visualization, recombinant genes were constructed to produce C-terminal GFP or RFP fusion proteins after a stronger promoter *H3* instead. Movma2-RFP and Movma11-GFP exhibited distribution patterns restricted to cellular structures which likely included vacuoles (Figure 2). When stained with the vacuolar dye CMAC during appressorium formation, fluorescence signals of both proteins showed good coincidence with the CMAC-positive vacuoles. However, there were some Movma11 resident compartments that could not be stained by CMAC (Figure 2). Further staining with DAPI indicated that these compartments were located around the nuclei (Figure 3). Besides, colocalization of Movma11-GFP with FM4-64 showed that Movma11 also resided on FM4-64 unstained structures in addition to vacuoles (Figure S1). In *N. crassa*, ER is considered to be composed of nuclear envelope as well as associated membranes [45], and it has been reported that ER and nuclear membranes of living plant cells are incapable of internalizing FM4-64 [46]. Taken together, these data revealed that both Movma2 and Movma11 were localized on vacuoles, while Movma11 was also distributed in a manner similar to ER localization. The distribution pattern of another GFP-tagged V_0_ domain subunit Movma16 was similar to that of Movma11-GFP (data not shown).

During appressorium development, Movma2-RFP colocalized with Movma11-GFP on the vacuoles, and the putative Movma11-anchored ER was possibly sequestered into the central vacuole in the end (Figure 3). Fluorescence signals eventually disappeared from the conidium (Figure 3, 24 h), suggesting the whole spore, including interior vacuoles and DAPI-stained nuclei, was collapsed during appressorium maturation.

### Characterization and disruption of the *VMA11* homologous gene in *M* *. oryzae*

*MoVMA11* (MGG_03065.7) putatively encodes small hydrophobic proteins (proteolipids, 168 amino acids long) with four transmembrane segments yet contains 4 introns, occupying about half of the DNA sequences of the gene. Amino acid sequences of Movma11 showed high identities to the homologs from other fungi (Figure S2A), such as *N. crassa* (85%) and *S. cerevisiae* (64%). Two V-ATPase proteolipid subunit c-like domains (IPR002379) were identified in Movma11, and the sequences were broadly conserved among fungi. Phylogenetic analysis revealed that vma11 proteins of pezizomycotina species are more closely related to each other than to those of other ascomycetes and basidiomycetes (Figure S2B).

To elucidate the role of V-ATPase complex during development and pathogenesis in *M* *. oryzae* , *MoVMA11* null mutants were generated through a targeted gene deletion strategy by replacing the *MoVMA11* ORF with the hygromycin-resistance cassette in the Guy11 WT background (Figure S3A). After initial locus-specific PCR screening, southern hybridization analysis was used to verify gene knockout mutants without random insertion by the detection of a single band shift from WT 5.1 kb to 8.9 kb (Figure S3B).

### *MoVMA11* is required for vacuolar acidification

**Figure 4:**
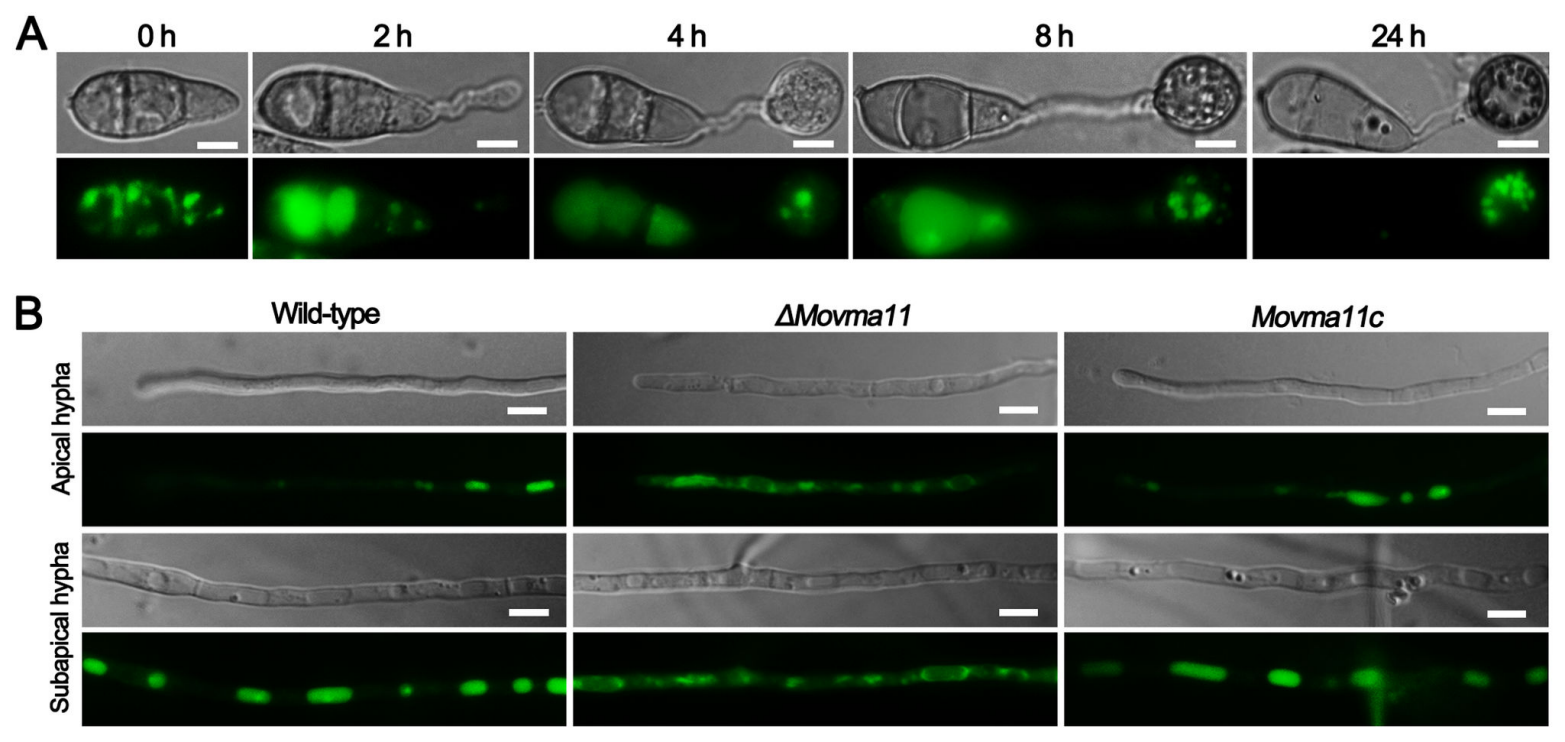
Quinacrine staning of acidic compartments in *M. oryzae*. (**A**) Observation of acidic compartments during appressorium development of WT strain. WT conidia were allowed to germinate on hydrophobic surfaces and stained by quinacrine at the indicated time points. Bars = 5 μm. (**B**) Disruption of *MoVMA11* disturbed vacuolar acidification. Hyphae of WT, *ΔMovma11*, and the complemented *Movma11c* strains were cultured on glass slides for 24 h, and then soaked in liquid CM supplemented with 200 μM quinacrine for 15 min. Compared with WT and *Movma11c* strains, vacuoles of the *ΔMovma11* mutant were less quinacrine-stained. Bars = 10 μm.

The pH status of *M* *. oryzae* vacuole during appressorium formation and vegetative growth was assessed with a pH-sensitive fluorescent dye, quinacrine, which can diffuse across membranes and accumulate in acidic compartments [40]. After incubating conidial suspension on the hydrophobic surfaces with different time points, WT vacuoles were quinacrine-stained by using 100 mM HEPES (pH 7.6) solution containing 200 μM quinacrine, and showed a distribution pattern highly similar to that of Movma2-RFP (Figure 4A). Fluorescent vacuoles were also observed in the vegetative hyphae of WT strain, whereas only some vacuolar membranes were visible in the *ΔMovma11* mutant under epifluorescence microscopy examination (Figure 4B). Expression of WT *MoVMA11* gene could rescue the mutant defect in vacuolar acidification (Figure 4B).

### Hyphal growth, asexual and sexual reproductions are dramatically impaired in the *ΔMovma11* mutant

**Figure 5:**
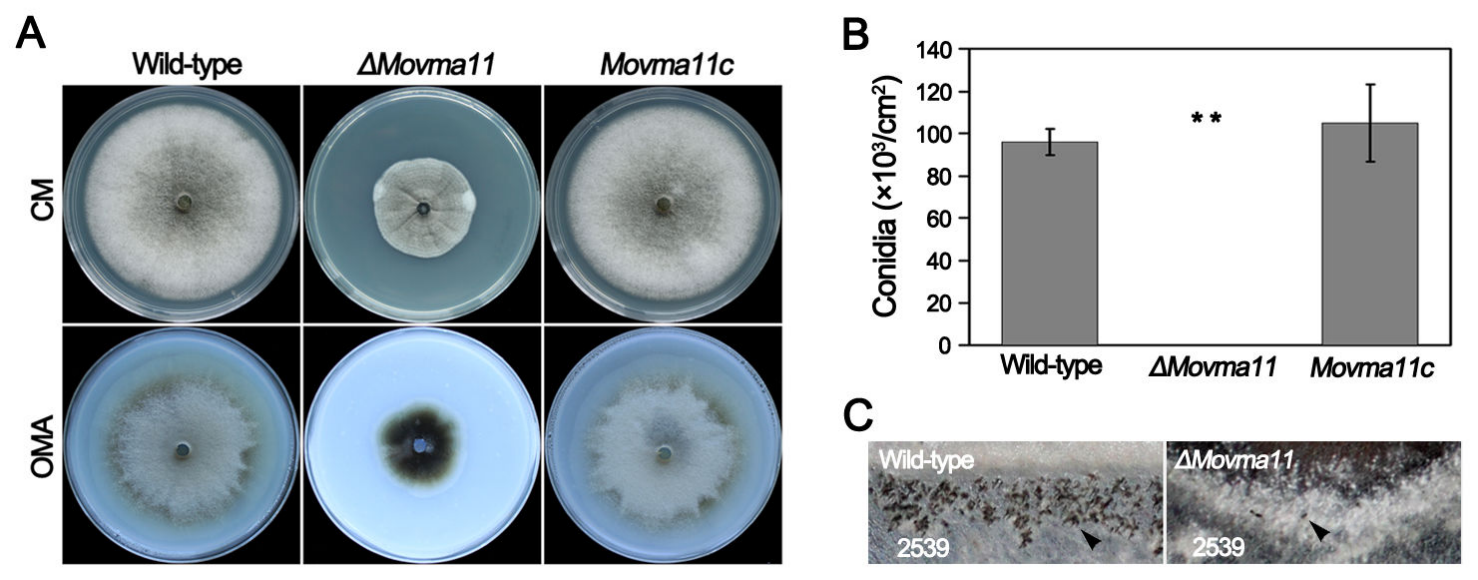
Severely impaired growth and conidiogenesis in the *ΔMovma11* mutant. (**A**) Colonies of the *ΔMovma11* mutant exhibited reduced appearances. Strains were incubated on CM or OMA agar plates for 12 d. (**B**) Conidia production of WT, *ΔMovma11*, and the complemented *Movma11c* strains. Data were collected 7 days postincubation on CM and presented as mean values and standard deviations derived from three independent experiments. Asterisks indicate significant differences among the tested stains. (**C**) Impaired sexual reproduction in the *ΔMovma11* mutant. Arrowheads indicate peritheria.

**Figure 6:**
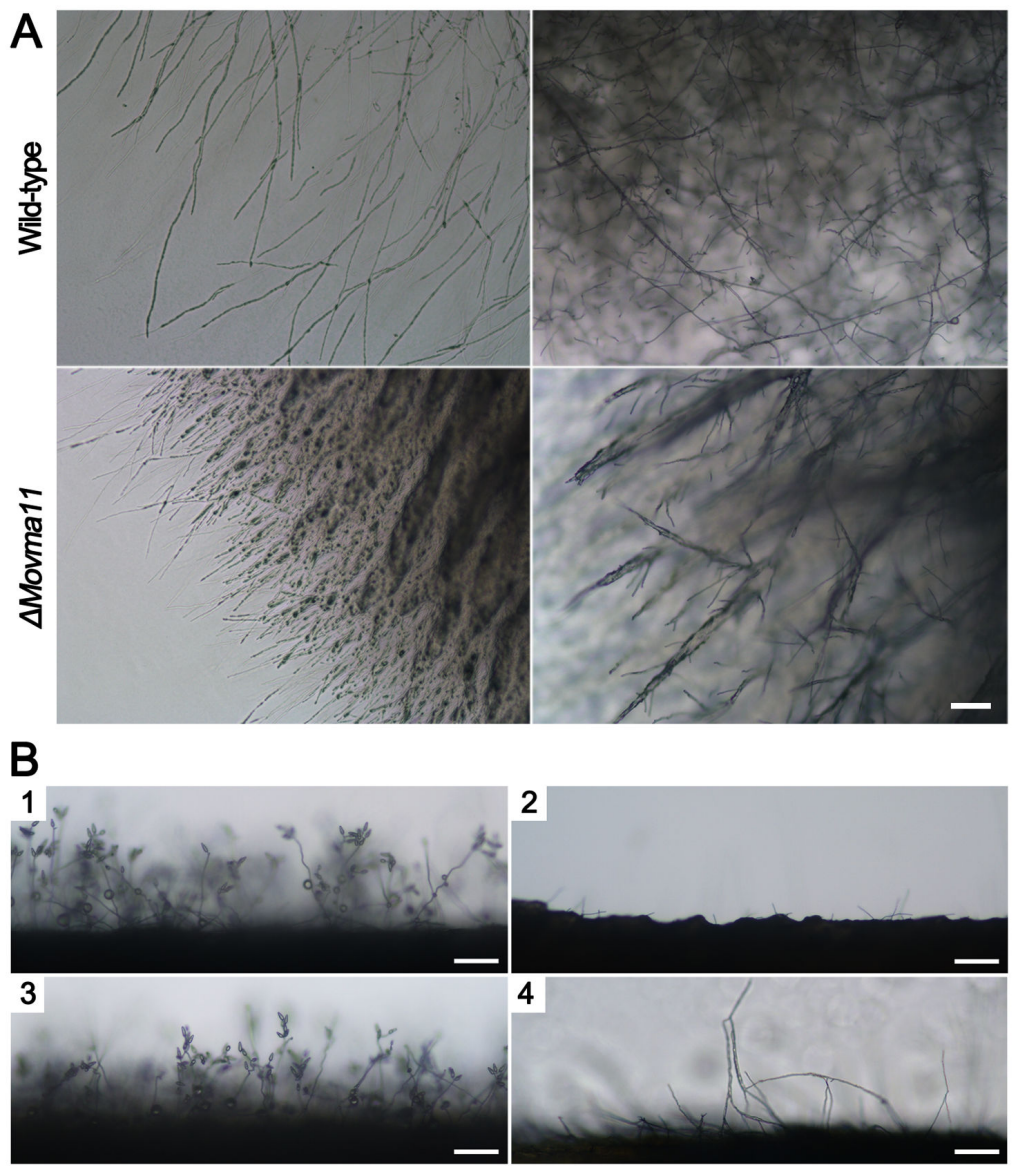
Hyphal growth and conidial development were observed under the microscope. (**A**) Microscopic examination of hyphal growth of WT and *ΔMovma11* strains. Pictures were zoomed in from 7-day-old cultures grown on CM plates. In contrast to WT stain, abnormal compact vegetative hyphae and badly entangled aerial hyphae were detected in the *ΔMovma11* mutant. (**B**) Aerial hyphal and conidial developments (**B1**). and (**B3**) Well developed conidia on conidiophores in WT and the complemented strains, respectively, at 24 hpi (**B2**). Few and very short aerial hyphae were developed by the *ΔMovma11* mutant, for the most part of the colony, at 24 hpi (**B4**). Long aerial hyphae, produced by the mycelia at colony margin of the *ΔMovma11* mutant, intertwined with each other at 48 hpi. Bars = 100 μm.

The effects of *MoVMA11* disruption on morphology and development were dramatic. The *ΔMovma11* mutant showed not only poor and restricted growth on medium, but also exhibited fewer aerial mycelia than WT or complemented strains (Figure 5A). Quantitative measurements confirmed that asexual sporulation was completely inhibited in the *ΔMovma11* mutant on CM or oatmeal agar plates (Figure 5B). For better visualization of the differences between WT and the deletion mutant in aerial hyphal and conidial development, microscopic examination was further performed with 7-day-old cultures grown on CM plates. Compared with WT, the *ΔMovma11* mutant showed a compact growth phenotype in which medium surfaces were crumpled and aerial hyphae were entangled into abnormal thick filamentous structures (Figure 6A). Subsequently, aerial hyphae were scraped away and mycelial agar blocks were kept under continuous illumination for conidiation. WT and complemented strains developed plenty of conidiophores with pyriform conidia sympodially arrayed at 24 hpi ( Figure 6B1 and 6B3, respectively). However, the *ΔMovma11* mutant, for the most part of the colony, formed rare and very short aerial hyphae, the length of which did not increase significantly even after prolonged incubation (Figure 6B2). Long aerial hyphae were produced only at the colony margin of the mutant, which were intertwined at 48 h hpi (Figure 6B4).

Studies of *N. crassa* reveal that *NcVMA11* deletion mutants have almost lost the ability to produce ascospores [14,47]. Sexual fertility of the *ΔMovma11* mutant was evaluated by crossing with the opposite mating-type strain 2539 after 4 weeks of incubation on oatmeal media. In contrast to the numerous perithecia and abundant asci developed by the WT and complemented strains, very few perithecia and no typical asci were observed in the *ΔMovma11* mutant (Figure 5C).

### Yeast Vma^−^-like phenotypes of the *ΔMovma11* mutant in carbon sources utilization, calcium and heavy metals sensitivities, but not in alkaline pH sensitivity

Growth of WT and *ΔMovma11* strains was tested on media with various carbon sources. The *MoVMA11* null mutant grew well on fermentable carbon sources (Table S3), but not on non-fermentable ones (Table 1). Among all the carbon sources tested, casein and triolein strongly affected the *ΔMovma11* mutant. In particular, the *ΔMovma11* mutant could not grow on medium with casein as carbon source. However, like WT strain, it could release extracellular enzymes degrading casein, forming a white halo surrounding the inoculation site (Figure S4 up panel).

**Table 1:** Effect of non-fermentable carbon sources on the diameter growth rate of the *ΔMovma11* mutant.

**Strain**	**Amylogen**	**Pectin**	**Olive oil**	**Triolein**	**Casein**
Guy11	104.9±3.0^A^	84.7±1.0^A^	85.6±3.4^A^	72.2±2.2^A^	60.2±2.7^A^
*ΔMovma11*	85.7±8.1^B^	69.5±1.8^B^	52.1±2.5^B^	17.4±3.1^B^	0^B^
*Movma11c*	108.3±1.7^A^	83.4±1.9^A^	87.3±1.0^A^	76.0±1.8^A^	54.3±3.0^A^

Diameter growth rate (%) = (the diameter of cultures on MM with different chemical/the diameter of regular MM cultures) × 100; colony sizes on regular MM are set as 100% control. Data were collected 7 days postincubation on media and presented as mean values and standard deviations from three independent experiments. Means followed by the same letter are not significantly different by Duncan’s multiple range tests at the 0.05 level of probability.

A low cytosolic concentration of Ca^2+^ and heavy metal cations is maintained by sequestering them into fungal vacuoles [48]. Growth of fungal *VMA* deletion mutants has been reported to be severely impaired by several ions [49,50]. When tested in *M* *. oryzae* , diameter growth rates of the *ΔMovma11* mutant were about the half of WT in the presence of Ca^2+^ (200 mM), Cu^2+^ (1 mM), and Fe^2+^ (3 mM). Mn^2+^ (3 mM) and Zn^2+^ (4 mM) were especially potent against the *ΔMovma11* mutant, which could hardly grow in the presence of these ions (Table 2).

**Table 2:** Effect of calcium and heavy metal cations on the diameter growth rate of the *ΔMovma11* mutant.

**Strain**	**0.2 M CaCl_2_**	**1 mM CuCl_2_**	**3 mM FeCl_2_**	**3 mM MnCl_2_**	**4 mM ZnCl_2_**
Guy11	54.7±3.4^A^	69.1±1.2^A^	92.0±3.6^A^	103.1±1.2^A^	44.7±9.2^A^
*ΔMovma11*	27.6±4.8^B^	29.5±4.9^B^	49.0±1.5^B^	2.7±3.0^B^	2.8±0.9^B^
*Movma11c*	52.6±2.8^A^	67.3±3.8^A^	96.4±1.7^A^	103.0±3.3^A^	40.4±3.1^A^

Diameter growth rate was determined as described in Table 1. Means with the same letter are not significantly different at the 0.05 level of probability according to Duncan’s multiple range tests.

To assess whether alkaline condition was toxic for the *ΔMovma11* mutant, mycelial agar plugs were incubated on MM or CM agar plates at different pH values. In contrast to the *VMA11* deletion mutant of *S. cerevisiae*, conditional lethality at alkaline pH was not found in the *ΔMovma11* mutant, the growth of which was almost the same at alkaline pH as acidic pH, except for fewer aerial hyphae (Figures S5A and S5B).

### Compromised appressorium formation and altered cell wall integrity in the *ΔMovma11* mutant

**Figure 7:**
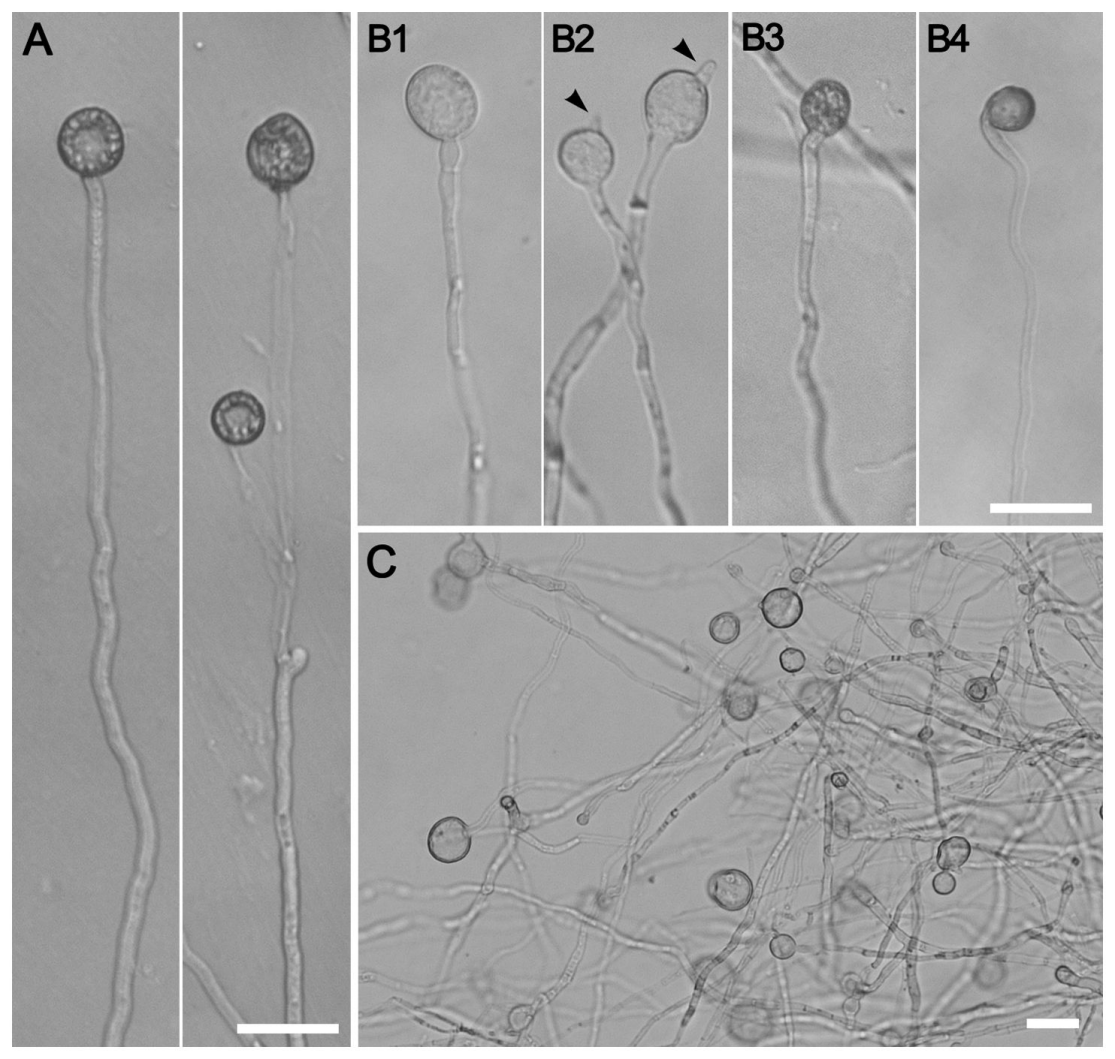
Appressorium development assays with hyphal tips on hydrophobic films. (**A**) Hyphal tips of WT formed healthy appressoria with numerous lipid droplets at 72 hpi (**B1-2**). Non-melanized swollen structures and (**B3-4**) small appressoria formed by the hyphal tips of the *ΔMovma11* mutant at 72 hpi (**B2**). Resumption of polarized growth and (**B4**) depletion of lipid droplets in some apical hyphal swellings and appressoria, respectively. Arrowheads indicate resumed polarized growth. (**C**) Abundant apical hyphal swellings were observed in the *ΔMovma11* mutant at 96 hpi. Bars = 20 μm.

To evaluate the role of *MoVMA11* in *M* *. oryzae* appressorium formation, growing hyphal suspension, harvested after 2 days shaking in liquid CM, was incubated on the inductive hydrophobic surfaces of plastic covers. Microscopic observation showed that WT strain formed numerous appressoria with plenty of lipid droplets remained 72 hpi at the hyphal tips (Figure 7A). Meanwhile, hyphal tips of the *ΔMovma11* mutant developed frangible swollen structures without melanization (Figure 7B1 and 7B2) or fully melanized appressoria that were extremely few (only several in number for each replicate) and smaller in size than WT or complemented strains (Figure 7B3 and 7B4). Some apical hyphal swellings resumed polarized growth (Figure 7B2), while intercalary hyphal swellings were hardly observed. At 96 hpi, apical hyphal swellings abounded (Figure 7C), but no obvious improvement in appressorial production was detected. Numerous swollen hyphal tips were also observed in the *ΔMovma11* mutant when incubating the mycelial suspension on non-inductive hydrophilic surfaces, but not when shaking the mycelial suspension gently.

Hyphal swelling indicated a cell wall defect of the *MoVMA11* disruption mutant [51]. Cell wall integrity of the *ΔMovma11* mutant was further examined by sensitivity assays with cell wall stressors, such as Calcofluor white (CFW) (200 μg/ml), Congo red (CR) (200 μg/ml), and SDS (0.01%). Differences between mycelial growth rates of WT and *ΔMovma11* strains were slight on CM agar with SDS or CFW, but were significant on CR-containing media (WT 46.3% vs. mutant 69.1%, *p*<0.05), also suggesting a role of *MoVMA11* in the maintenance of cell wall integrity.

### *MoVMA11* is essential for pathogenicity

**Figure 8:**
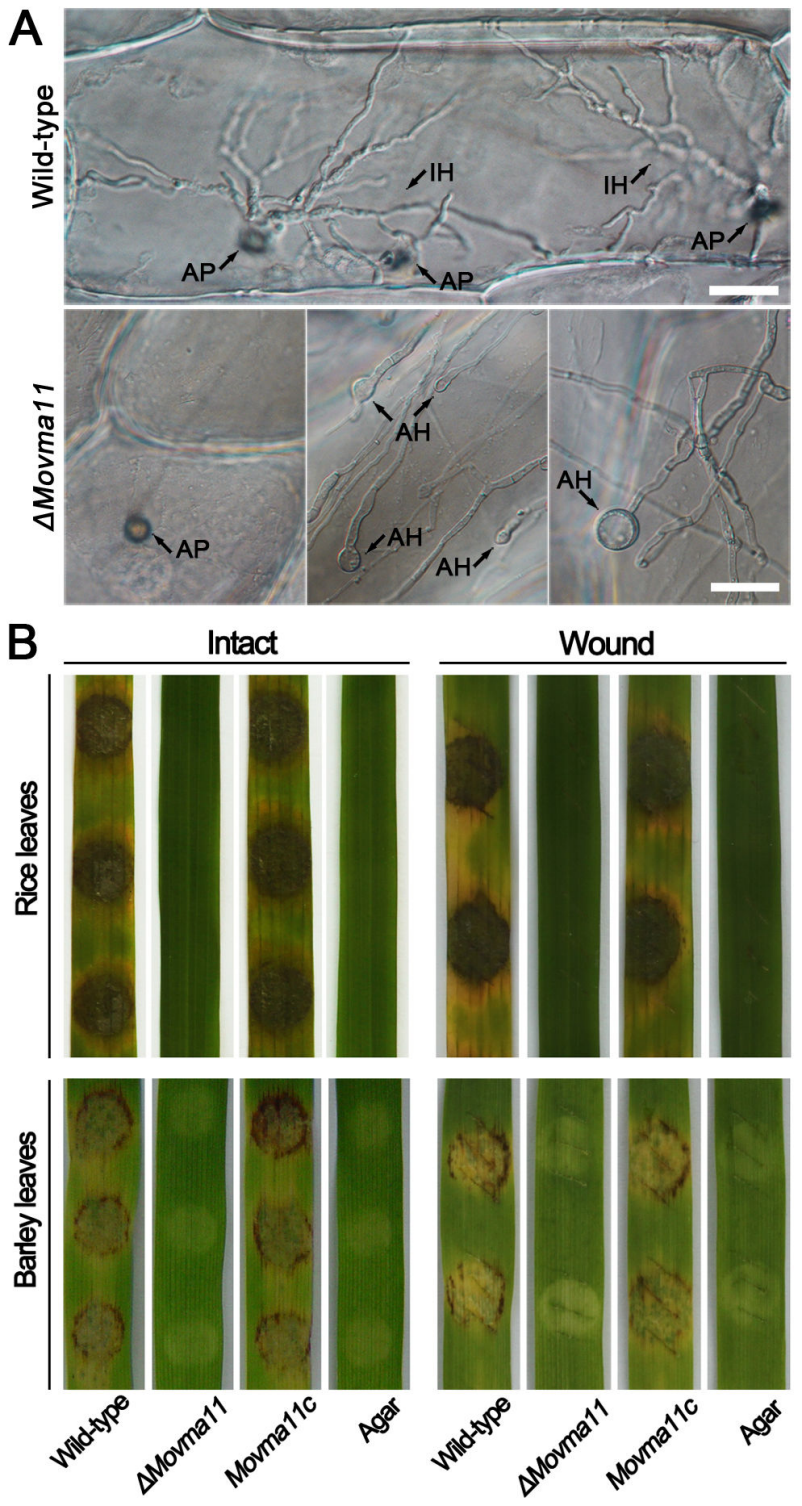
*MoVMA11* is essential for pathogenicity. (**A**) In penetration assays with onion epidermal cells, rare appressoria formed by *MoVMA11* disruption mutant failed to penetrate or grow invasively in cells, and apical hyphal swellings were also frequently observed in the *ΔMovma11* mutant on the epidermal cell surfaces. Bars = 25 μm. AP, appressorium; IH, invasive hyphae; AH, apical hyphal swelling. (**B**) Pathogenicity assays on intact and wounded host plant leaves. Rice and barely leaves, incubated with mycelial agar plugs of tested strains, were elevated 5 and 4 days after incubation, respectively.

Due to the conidiation failure of the *ΔMovma11* mutant, mycelial agar plugs, rather than conidial suspension, were inoculated onto susceptible rice or barley leaves to perform a pathogenicity assay. The *ΔMovma11* mutant failed to infect the hosts and cause the same blast lesions as the WT and complemented strains did (Figure 8B left panel). To define the specific drawbacks of the *MoVMA11* null mutant in appressorial penetration and/or invasive growth, penetration on onion epidermal cells was assayed with mycelial suspensions (Figure 8A). In agreement with what observed on artificial film surfaces, swollen structures and smaller appressoria were produced at the hyphal tips of the *ΔMovma11* mutant. In contrast to the extensive growth of invasive hyphae of WT at 72 hpi, the appressoria formed by the *ΔMovma11* mutant failed to develop penetration pegs or infectious hyphae in plant cells under the same condition, or even after prolonged incubation time.

Because surface incubation of the *ΔMovma11* mutant failed to cause any host lesions, wounded rice and barley leaves were employed to determine whether the *ΔMovma11* mutant was capable of invasive growth *in planta*. Like the pathogenicity assays on intact leaves, the *ΔMovma11* mutant was unable to elicit any necrosis at the wounded sites of host tissues (Figure 8B right panel).

## Discussion

Eukaryotic V-ATPase is associated with numerous cellular functions [8], and the virulence of human pathogenic fungi, *C. albicans* and *Cryptococcus neoformans*, also requires functional V-ATPase [13,52]. In this study, we set out to explore the role of V-ATPase complex in the development and pathogenicity of the plant pathogenic fungus *M* *. oryzae* .

Three V-ATPase subunits, Movma11, Movma16, and Movma2, were tagged with green or red fluorescent proteins to determine the subcellular location of V_1_ and V_0_ domains in *M* *. oryzae* . According to the yeast GFP fusion localization database (http://yeastgfp.yeastgenome.org/) [53], Scvma11 is ER-resident whereas Scvma16 is vacuole/ER-resident, although epitope-tagged Scvma11 and Scvma16 are detected on the vacuolar membrane [54]. Our results indicated that both of the tested GFP-tagged subunits of V_0_ domain, Movma11 and Movma16, exhibited a similar distribution pattern on vacuole and putative ER as Scvma16-GFP. Lumenal orientation of Movma11 and Movma16 C-termini [55] and normal internalization of vacuolar membranes may be the reasons that lead to the lumenal distribution pattern of fusion proteins in vacuoles. Moreover, a putative ER-retained fluorescence signal was observed possibly due to that V_0_ domain is assembled in ER before its delivery to the destination [7]. Unlike the subunits of V_0_ domain, V_1_ domain subunit vma2 has no membrane spanning region and lies on the cytosolic side of the membrane [9], thus Movma2-RFP signal was detected predominantly in the vacuolar membrane of large vacuoles. Further studies indicated that disruption of one such subunit, Movma11, resulted in pleiotropic effects on the growth and development of *M* *. oryzae* .

The *MoVMA11* null mutant exhibited yeast Vma^-^-like phenotypes. The vacuolar detoxification ability relies on an acidic lumen to build up the proton gradient as the motive force for toxic ions trafficking against their gradient [56]. The *ΔMovma11* mutant was defective in vacuolar acidification, and thus highly sensitive to Ca^2+^ and sorts of heavy metal cations. Compared to fermentable substrates, significant reductions in diameter growth rates of the *ΔMovma11* mutant were also detected on media supplemented with various non-fermentable substrates as the sole carbon sources. The *ΔMovma11* mutant failed to grow on casein, but formed a white halo surrounding the inoculation site, indicating the secretion of extracellular depolymerizing enzymes. Besides, the deletion mutant showed a smaller yet darker appearance than WT in the laccase activity assay (Figure S4 down panel). Thus, the drawbacks of the *ΔMovma11* mutant in non-fermentable substrate usage might not be related to exocytosis, or only a portion of extracellular enzymes required Movam11 for secretion, similar to the role of P4-type ATPase *MoAPT2* in exocytosis [57].

Nevertheless, differences still exit among fungi. It has been reported that the growth of *vma* mutants in *S. cerevisiae* can be restored by iron ions [49], but supplementation with Fe^2+^ caused a further reduction, rather than improvement, in the growth rate of the *ΔMovma11* mutant, similar to the results reported for *VMA* disruptants in *A. nidulans* [58] and *A. niger* [59]. Another compound inositol, also reported to stimulate the growth of *S. cerevisiae vma* mutants [11], failed to suppress the Vma^-^ phenotype in the *ΔMovma11* mutant as well as *N. crassa vma1 mutants* [14]. Most surprisingly, while yeast and many other fungi, including *C. albicans* [13], *S. pombe* [12], and *A. oryzae* [60], exhibit a characteristic pH-dependent growth phenotype upon disruption of V-ATPase function, however, the *ΔMovma11* mutant did not show conditional lethality at alkaline pH, the same as the *N. crassa vma11* mutant [47], *Ashbya* *gossypii* *vma1* mutant [61], and *A. niger* conditional *vma6* mutant [59]. Like proposed in the *ΔNcvma11* mutant, *M* *. oryzae* strain lacking subunit c’ may also retain some V-ATPase activities, which aided the strain in survival at alkaline pH condition. The reasons could be glimpsed from the special evolutional mode of subunit c’, and the high similarities between subunits c and c’. Subunit c’ is only present in fungi, but not in other organisms [47]. A recent elegant research by Finnigan et al. [5] indicates that vma3 and vma11 are sister proteins duplicated from the same ancestral gene, and differences occur when the ancestors lose their flexibility of interaction interfaces; it is also observed that no extra functions are gained by the modern yeast vma3 and vma11 after the duplication, and single amino-acid substitution in ancestors could change their capacities to complement modern *VMA* gene deletions, possibly by influencing interaction interfaces. Since there are many amino-acid differences between protein sequences of Scvma3 and Movma3/Ncvma3, divergences may occur during the evolution so that subunit c retain some abilities of substitution for subunit c’ in *M* *. oryzae* or *N. crassa* [47].

Cell wall structure and function are affected in *S. cerevisae vma* mutants and the recently reported *vma6* mutant of *Aspergillus niger* [59,62]. *MoVMA11* is also involved in cell wall integrity, as the null mutant was more resistant to cell wall perturbing agent CR and formed abundant swollen hyphal tips on film surfaces. Apical hyphal swelling is likely due to the weakened cell wall with disturbed formation of melanin layer and increased internal turgor after surface sensing. Previous reports indicate that the cAMP signaling pathway plays a role in the regulation of melanin synthesis among fungal pathogens *C. neoformans* [63], *A. fumigatus* [64], and *Ustilago* *hordei* [65], and full activation of the cAMP/PKA pathway requires a functional V-ATPase [4]. Our data showed that addition of exogenous cAMP caused darker pigmentation during mycelial growth on MM (Figure S5C) and effectively inhibited the production of large swollen structures at the hyphal tips of the *ΔMovma11* mutant on film surfaces (data not shown). Therefore, disruption of *MoVMA11* might decrease melanin biosynthesis or deposition of cell wall through the cAMP/PKA pathway under exogenous nutrient-poor conditions, such as growth on MM or appressorium formation. Weakened cell wall might to some extent be the reason why aerial hyphae of the *ΔMovma11* mutant intertwined with each other and displayed a low vitality, as tested by culturing scraped aerial hyphae in liquid CM (data not shown). However, suppression of apical hyphal swelling could be a result from the osmotic effect of exogenous 10 mM cAMP. Further studies are to be carried out to demonstrate conclusively the relationships among V-ATPase, melanin biosynthesis, and cAMP/PKA signaling pathway.

For successful colonization and further reproduction in host plants, *M* *. oryzae* and other phytopathogenic fungi require a large variety of morphogenetic and metabolic processes to rupture the plant cuticle and then overcome the fierce defenses of the plants [66]. It has been reported that compared with non-pathogenic fungi, gene families predicted to be components of V-ATPase complex are contracted in phytopathogenic fungi, including *M* *. oryzae* [67]. Thus, it is reasonable that vacuoles in pathogenic fungi, the centre of networks enabling various physiological processes, may be regulated by V-ATPase in a simpler way than their non-pathogenic relatives, and maintain long-term acidic and active during infection-related developments. Indeed, we observed that the *M* *. oryzae* vacuoles were acidic as visualized by quinacrine staining during appressorium formation. Besides, our colocalization analyses also showed that the V-ATPase V_1_ domain subunit was coupled with that of the V_0_ domain, indicative of fully assembled and functional V-ATPase holoenzymes in appressorial development (Figure 2) and invasive growth (Figure S6). However, disruption of *MoVMA11* reduced vacuolar acidification. On the other hand, V_0_ domain of V-ATPase is also involved in membrane fusion [68,69]. Recently, vacuolar fusion protein Momon1 [34] and soluble NSF attachment protein receptor (SNARE) protein Movam7 [70], both of which participate in vacuolar membrane trafficking, are shown to be involved in conidiogenesis and appressorium development. Hence, severely defeats of the *ΔMovma11* mutant in vegetative growth, asexual and sexual reproductions, appressorium formation, and host infection might be the results of abnormal cellular storage, turnover or membrane dynamics due to impaired vacuolar acidification and the proteolipid ring of the V_0_ domain. Possibly connected to the regulation of melanin biosynthesis or deposition through cAMP signal transduction, the *MoVMA11* null mutant also showed disturbed cell wall integrity. Taken together, the V-ATPase c’ subunit Movma11 is crucial for various patterns of growth and development in *M* *. oryzae* , and this study provides the foundation for further research on V-ATPase and intracellular pH regulation in *M* *. oryzae* and other phytopathogenic fungi.

## Supporting Information

Figure S1 FM4-64 staining of Movma11-GFP-expressing strain. Conidia of Movma11-GFP-expressing strain were incubated with 7.5 μM FM4–64 and allowed to germinate on hydrophobic surfaces for 2 h before the solution was gently substituted by sterile water. FM4–64 was used to stain vacuoles and endocytic compartments. Development of appressorium was observed at the indicated time points. Arrowheads indicate FM4-64-unstained structures that Movma11 anchored. Bars = 5 μm.
(TIF)

Figure S2 Amino acid sequence alignment and phylogenetic analysis of vma11 proteins in fungi. (**A**) Protein sequence alignment of Movma11 with its fungal homologs. Sequences were aligned with ClustalW2 program (http://www.ebi.ac.uk/Tools/msa/clustalw2/). V-ATPase proteolipid subunit c-like domain (IPR002379) was predicted by InterPro (EMBL-EBI) based on Movma11 protein sequence. Identical and similar residues are shown by black or gray backgrounds, respectively. Compared proteins are from *Magnaporthe* *oryzae* (MGG_03065.7), *Saccharomyces cerevisiae* (NP_015090.1), *Candida albicans* (XP_721376.1), *Yarrowia lipolytica* (XP_504637.2), *Aspergillus niger* (XP_001391591.1), *Coccidioides* *posadasii* (XP_003070013.1), *Cochliobolus* *sativus* (EMD63759.1), *Sclerotinia sclerotiorum* (XP_001595091.1), *Neurospora crassa* (XP_965807.1), *Gibberella* *zeae* (XP_388749.1), *Trichoderma* *reesei* (EGR46584.1), *Fusarium oxysporum* (EGU77702.1), *Schizosaccharomyces pombe* (NP_593600.1), and *Cryptococcus neoformans* (AFR92415.1). (**B**) Phylogenetic analysis of V-ATPase subunit c’ in fungi. Mega5.1 program was used for phylogenetic tree construction by the neighbor-joining method with 1000 bootstrap replicates. Numbers at each node indicate bootstrap values (percentage of 1000 replicates).
(TIF)

Figure S3 Targeted gene replacement strategy. (**A**) The *MoVMA11* gene deletion vector was constructed by double-joint PCR. The orientations and positions of primers VMA11up-1/2, VMA11dn-1/2, HPH-1/2, and nVMA11-1/2 are indicated as 1-8, respectively, with small arrows. Sa = *Sac*I, P = *Pst*I, S = *Sal*I. (**B**) Southern blot analysis of *MoVMA11* deletion transformants. *Sac*I-digested genomic DNAs were hybridized with a probe amplified with primers VMA11pb-1/2. As expected, 5.1 kb and 8.9 kb bands were observed in WT strain and two *ΔMovma11* mutants, respectively.
(TIF)

Figure S4 Exocytosis-related assays in *MoVMA11* disrutionp mutant. Up panel showed the growth of WT and *ΔMovma11* strains on MM using casein as carbon source. The white halo formed by the incubation of the *ΔMovma11* mutant was indicated by arrowhead. Down panel was the assay for extracellular laccase activity. Strains were incubated on CM supplemented with 0.2 mM ABTS [2, 2’-azino-di(3-ethylbenzthiazoline-6-sulfonate)] for 3 days before photography.
(TIF)

Figure S5 Growth of WT and *ΔMovma11* strains on media at different pH or supplemented with cAMP. (**A**) MM and (**B**) CM agar plates added with 20 mM HEPES, adjusted to pH 5.6-8.2, were used to culture strains for 7 days. No significant differences were found between the growths of *ΔMovma11* mutants at alkaline pH and acidic pH. (**C**) The *ΔMovma11* mutant showed a darker pigmentation in response to exogenous cAMP. Pictures were taken 8 days after inoculation of agar plugs on MM agar plates.
(TIF)

Figure S6 Colocalization analysis of Movma11-GFP and Movma2-RFP during infectious growth. Movma11 of V_1_ domain was coupled with Movma2 of V_0_ domain in invasive hyphae. Conidial suspension of strain expressing both Movma11-GFP and Movma2-RFP was inoculated on onion epidermal cells for 65 h before photography. Bars = 25 μm.
(TIF)

Table S1 List of primers in this study. (DOC)

Table S2 Characteristics of V-ATPase subunits and the putative homologs in *M* *. oryzae* . (DOC)

Table S3 Effect of fermentable carbon sources on the diameter growth rate of the *ΔMovma11* mutant. (DOC)
