# Supplementary figures and images for: Involvement of MoVMA11, a Putative Vacuolar ATPase c’ Subunit, in Vacuolar Acidification and Infection-Related Morphogenesis of Magnaporthe oryzae
Source: PLoS One. 2013 Jun 27;8(6):e67804. doi: 10.1371/journal.pone.0067804 (PMC3694887; doi:10.1371/journal.pone.0067804)

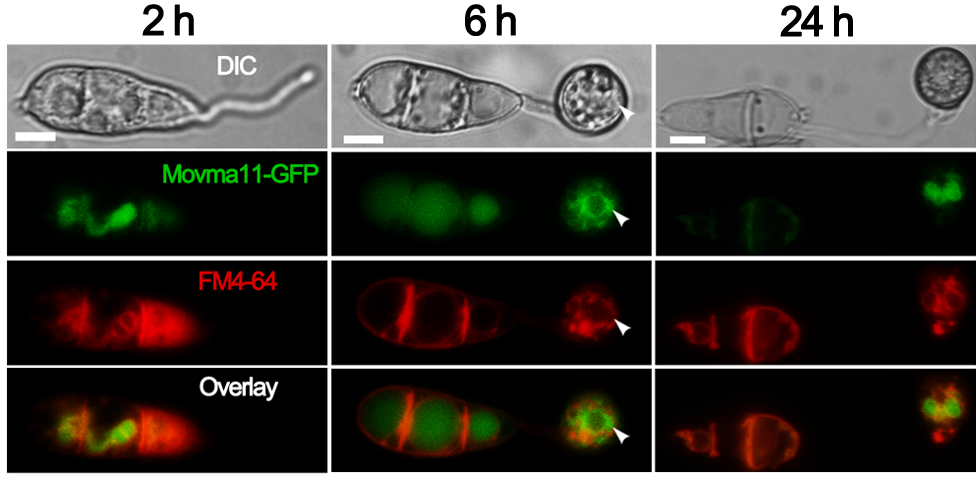

Supplement: Figure S1 — Conidia of Movma11-GFP-expressing strain were incubated with 7.5 μM FM4–64 and allowed to germinate on hydrophobic surfaces for 2 h before the solution was gently substituted by sterile water. FM4–64 was used to stain vacuoles and endocytic compartments. Development of appressorium was observed at the indicated time points. Arrowheads indicate FM4-64-unstained structures that Movma11 anchored. Bars = 5 μm. (TIF) [file pone.0067804.s001.tif]

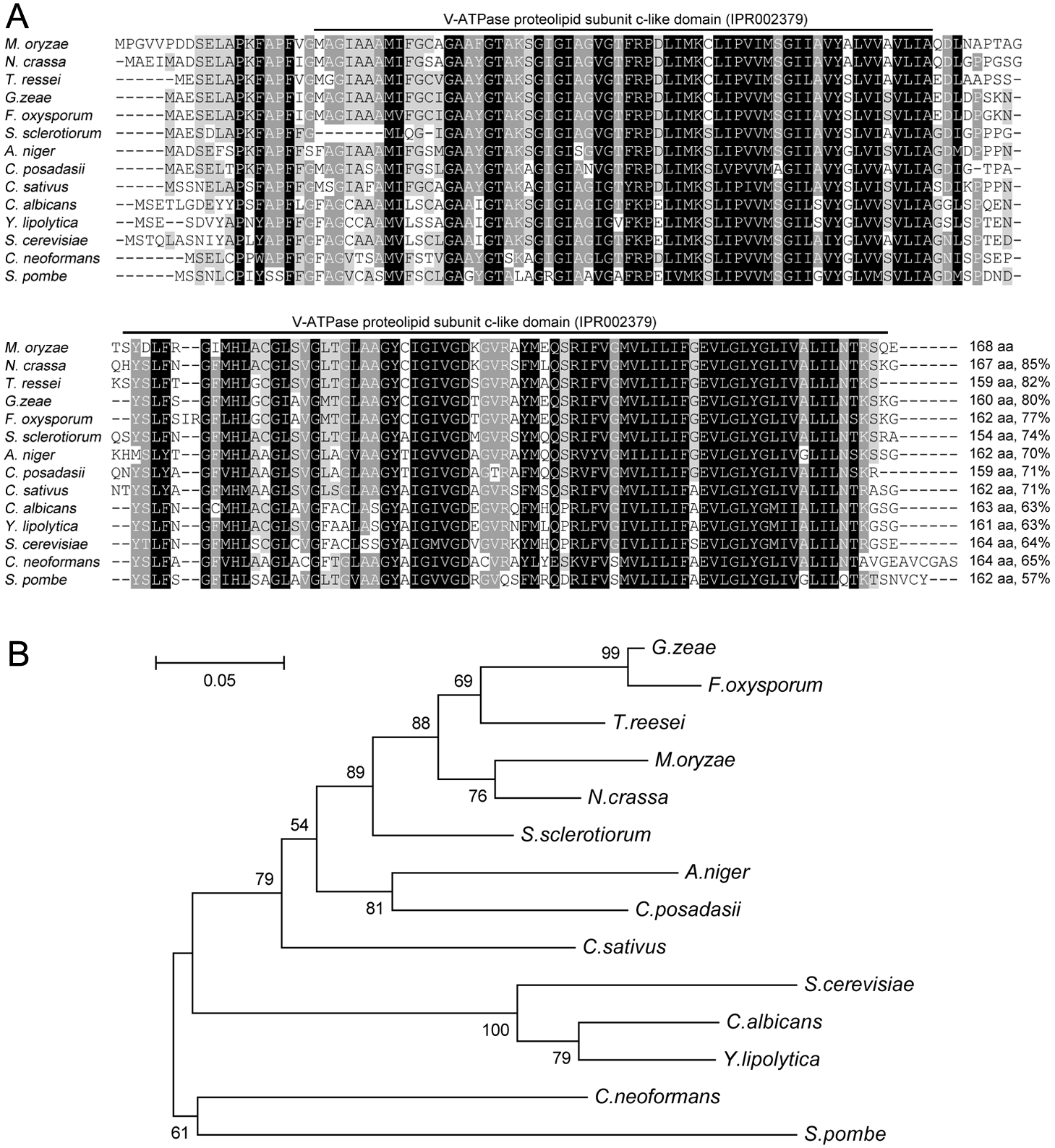

Supplement: Figure S2 — (A) Protein sequence alignment of Movma11 with its fungal homologs. Sequences were aligned with ClustalW2 program (http://www.ebi.ac.uk/Tools/msa/clustalw2/). V-ATPase proteolipid subunit c-like domain (IPR002379) was predicted by InterPro (EMBL-EBI) based on Movma11 protein sequence. Identical and similar residues are shown by black or gray backgrounds, respectively. Compared proteins are from Magnaporthe oryzae (MGG_03065.7), Saccharomyces cerevisiae (NP_015090.1), Candida albicans (XP_721376.1), Yarrowia lipolytica (XP_504637.2), Aspergillus niger (XP_001391591.1), Coccidioides posadasii (XP_003070013.1), Cochliobolus sativus (EMD63759.1), Sclerotinia sclerotiorum (XP_001595091.1), Neurospora crassa (XP_965807.1), Gibberella zeae (XP_388749.1), Trichoderma reesei (EGR46584.1), Fusarium oxysporum (EGU77702.1), Schizosaccharomyces pombe (NP_593600.1), and Cryptococcus neoformans (AFR92415.1). (B) Phylogenetic analysis of V-ATPase subunit c’ in fungi. Mega5.1 program was used for phylogenetic tree construction by the neighbor-joining method with 1000 bootstrap replicates. Numbers at each node indicate bootstrap values (percentage of 1000 replicates). (TIF) [file pone.0067804.s002.tif]

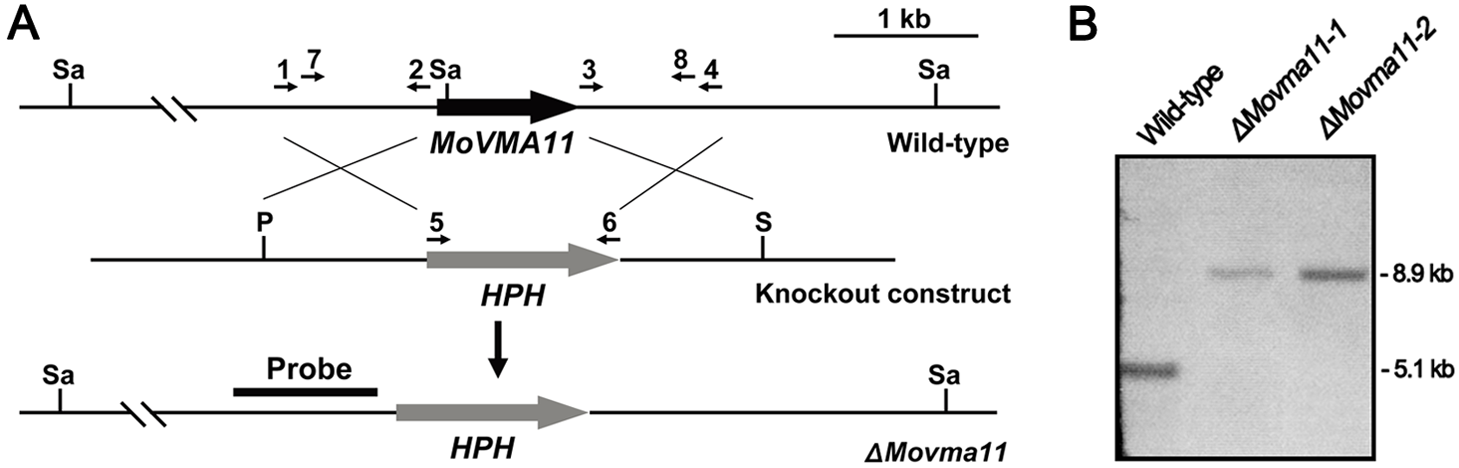

Supplement: Figure S3 — (A) The MoVMA11 gene deletion vector was constructed by double-joint PCR. The orientations and positions of primers VMA11up-1/2, VMA11dn-1/2, HPH-1/2, and nVMA11-1/2 are indicated as 1-8, respectively, with small arrows. Sa = SacI, P = PstI, S = SalI. (B) Southern blot analysis of MoVMA11 deletion transformants. SacI-digested genomic DNAs were hybridized with a probe amplified with primers VMA11pb-1/2. As expected, 5.1 kb and 8.9 kb bands were observed in WT strain and two ΔMovma11 mutants, respectively. (TIF) [file pone.0067804.s003.tif]

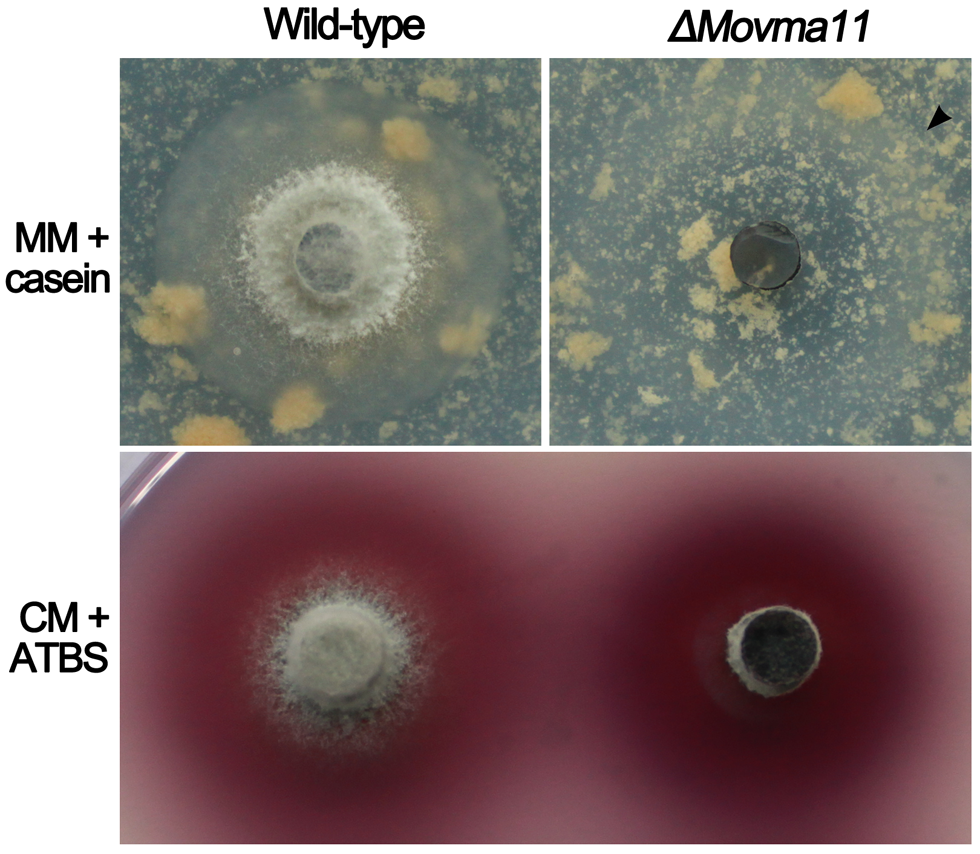

Supplement: Figure S4 — Up panel showed the growth of WT and ΔMovma11 strains on MM using casein as carbon source. The white halo formed by the incubation of the ΔMovma11 mutant was indicated by arrowhead. Down panel was the assay for extracellular laccase activity. Strains were incubated on CM supplemented with 0.2 mM ABTS [2, 2’-azino-di(3-ethylbenzthiazoline-6-sulfonate)] for 3 days before photography. (TIF) [file pone.0067804.s004.tif]

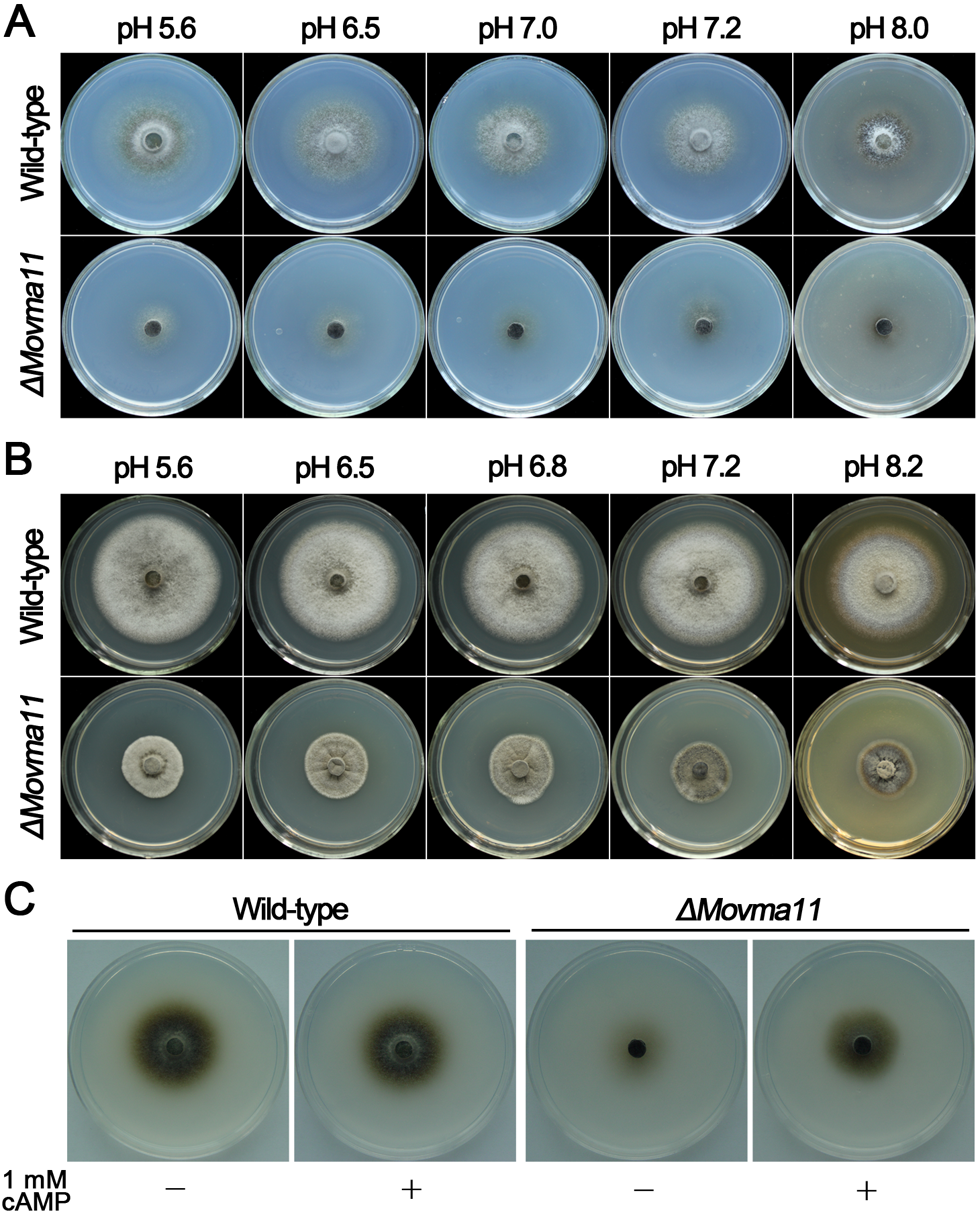

Supplement: Figure S5 — (A) MM and (B) CM agar plates added with 20 mM HEPES, adjusted to pH 5.6-8.2, were used to culture strains for 7 days. No significant differences were found between the growths of ΔMovma11 mutants at alkaline pH and acidic pH. (C) The ΔMovma11 mutant showed a darker pigmentation in response to exogenous cAMP. Pictures were taken 8 days after inoculation of agar plugs on MM agar plates. (TIF) [file pone.0067804.s005.tif]

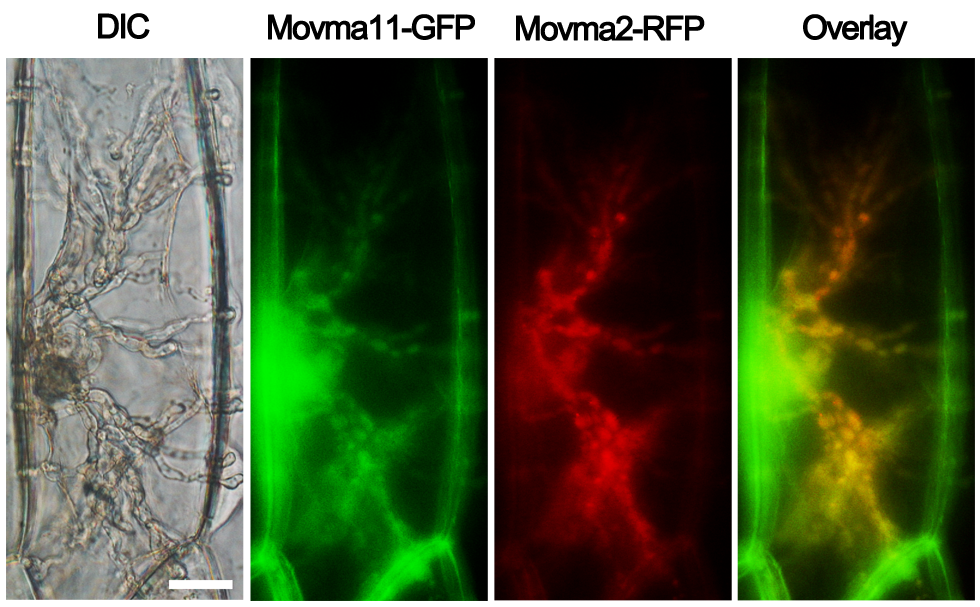

Supplement: Figure S6 — Movma11 of V1 domain was coupled with Movma2 of V0 domain in invasive hyphae. Conidial suspension of strain expressing both Movma11-GFP and Movma2-RFP was inoculated on onion epidermal cells for 65 h before photography. Bars = 25 μm. (TIF) [file pone.0067804.s006.tif]
