# Supplementary material for: Involvement of MoVMA11, a Putative Vacuolar ATPase c’ Subunit, in Vacuolar Acidification and Infection-Related Morphogenesis of Magnaporthe oryzae
Source: PLoS One. 2013 Jun 27;8(6):e67804. doi: 10.1371/journal.pone.0067804 (PMC3694887; doi:10.1371/journal.pone.0067804)
Supplement: Table S1 — (DOC) [file pone.0067804.s007.doc]

**Table S1.** List of primers used in this study.

| **Name** | **Sequence (5’ - 3’)** |
| --- | --- |
| VMA11up-1 | CGACGCCAGTATCTTGTTTA |
| VMA11up-2 | CATTCATTGTTGACCTCCACTAACGTGATTTATTGCAGCTGCC |
| VMA11dn-1 | GGGCAAAGGAATAGAGTAGATGAGGAATAATTCGGACCTGTCG |
| VMA11dn-2 | GGGACCCAATCCTGAAACTG |
| HPH-1 | TAGTGGAGGTCAACAATGAATG |
| HPH-2 | CATCTACTCTATTCCTTTGCCC |
| nVMA11-1 | AACTGCAGGCAGGTAGTGGGTATAGACGC |
| nVMA11-2 | CAGTCGACAGACTTTCAGCCTCGCAATAG |
| Vma11-C1 | CCGGAGCAATGATAAGAAAT |
| Vma11-C2 | CATACTAGCACTAGGGAACACG |
| VMA11pb-1 | GTAGTGGGTATAGACGCTGGAA |
| VMA11pb-2 | GAATGTCGTCGTTCTGTTGG |
| H3-1 | TTGATTCAGTCATGTTGATTGAGGTGTTGT |
| H3-2 | TGTCTAGACTTCCCGGGGATGGATCCGGCCATTGTGATTGATTTGTGATT |
| eGFP-1 | AACCCGGGATGGTGAGCAAGGGCGAGGAG |
| eGFP-2 | AATCTAGACTTGTACAGCTCGTCCATGCCG |
| SUR-1 | AACTCGAGGTGCCAACGCCACAGTGCC |
| SUR-2 | GCGAATTCACTAGTGATTGTGAATCGTGAGAGCATGCAATTCCC |
| DsRED-1 | AACCCGGGATGGCCTCCTCCGAGAACG |
| DsRED-2 | CGCTCTAGACAGGAACAGGTGGTGGCGG |
| NEO-1 | AACTCGAGGAGGTCAACACATCAATGCTA |
| NEO-2 | CGACTAGTTCAGAAGAACTCGTCAAGAAG |
| VMA11N-1 | CGGGATCCATGCCAGGAGTAGTACCGGA |
| VMA11N-2 | ATCCCGGGTTCCTGGCTCCTGGTGTTG |
| VMA16N-1 | AGCCCGGGATGAGCTTAACTTATGGACTCGG |
| VMA16N-2 | AGCCCGGGTCCACTGGATCCAAAGTCATTG |
| VMA2N-1 | AACCCGGGATGGCGGACCCTCGAGAGTC |
| VMA2N-2 | CACCCGGGGGCATCGATCAAGTTTTCCTCC |
|  |  |
| qRT-PCR |  |
| VMA2-QF | CTGTATGCAAAGTACGCCATTG |
| VMA2-QR | TTGTCTTCAGCCGATAGTGC |
| VMA4-QF | GCAAGGCCGATTATGACATTG |
| VMA4-QR | AGAATAGCAACACCGCCAG |
| VMA5-QF | CTACGCTCTGGTTTCTTTGC |
| VMA5-QR | AGGTATTGTGAAGGGTTGGAC |
| VPH1-QF | GTGTTCTTTGTGTTGAGCGTC |
| VPH1-QR | TGAGTGAAAGAGAATGGCTGG |
| VMA3-QF | GCTGCCATTGTCTTCACTTG |
| VMA3-QR | GCCATAATGACGGGAACAATG |
| VMA11-QF | CGGTCTTACTGGTCTTGCTG |
| VMA11-QR | CCGAAAATCAGAATCAGCACC |
| VMA16-QF | CTTCCAGGCCAAAATCAACAC |
| VMA16-QR | CCATTGATTCCAACAGCAACG |
| β-Tubulin-QF | ACAACTTCGTCTTCGGTCAG |
| β-Tubulin-QR | GTGATCTGGAAACCCTGGAG |
