# Supplementary material for: Involvement of MoVMA11, a Putative Vacuolar ATPase c’ Subunit, in Vacuolar Acidification and Infection-Related Morphogenesis of Magnaporthe oryzae
Source: PLoS One. 2013 Jun 27;8(6):e67804. doi: 10.1371/journal.pone.0067804 (PMC3694887; doi:10.1371/journal.pone.0067804)
Supplement: Table S2 — (DOC) [file pone.0067804.s008.doc]

**Table S2.** Characteristics of V-ATPase subunits and the putative homologs in *M. oryzae*

| Sturcture | Function | Subunit | Yeast gene | *M. oryzae* hits (identity) | Protein sequence analysis and classification |
| --- | --- | --- | --- | --- | --- |
| A_3_B_3_ hexamer | ATP hydrolysis | A | *VMA1* | MGG_08087 (42%) | 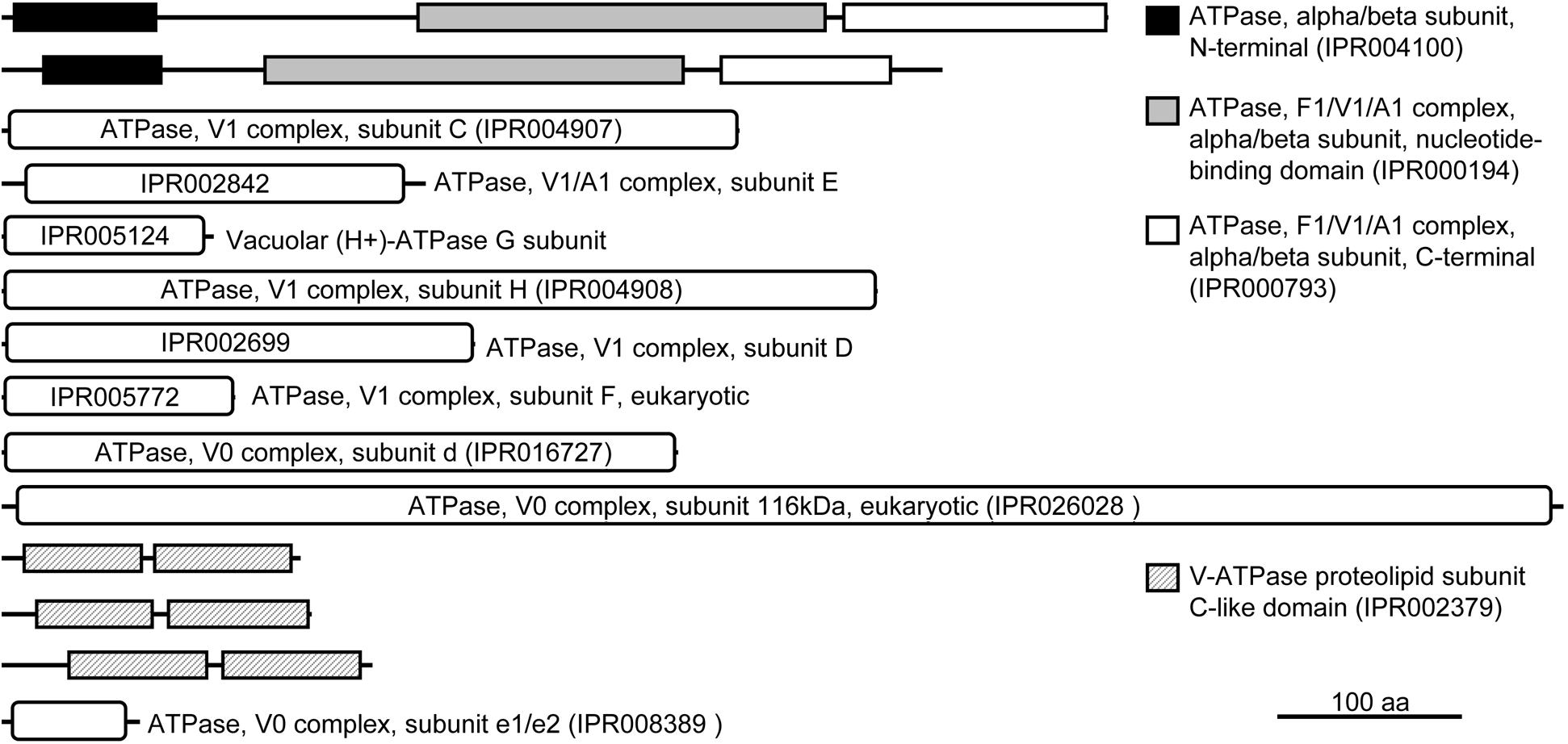 |
|  |  | B | *VMA2* | MGG_03244 (78%) |  |
| Peripheral stalk | Stator | C | *VMA5* | MGG_06349 (35%) |  |
|  |  | E | *VMA4* | MGG_02770 (43%) |  |
|  |  | G | *VMA10* | MGG_04716 (37%) |  |
|  |  | H | *VMA13* | MGG_04827 (17%) |  |
| Central stalk | Rotor | D | *VMA8* | MGG_07086 (68%) |  |
|  |  | F | *VMA7* | MGG_01556 (60%) |  |
|  |  | d | *VMA6* | MGG_07007 (53%) |  |
|  | Stator, proton pore | a | *VPH1/STV1* | MGG_03947 (45/43%) |  |
| Proteolipid ring | Rotor, proton pore | c | *VMA3* | MGG_02466 (77%) |  |
|  |  | c’ | *VMA11* | MGG_03065 (64%) |  |
|  |  | c’’ | *VMA16* | MGG_06108 (56%) |  |
|  | Unknown | e | *VMA9* | MGG_01505 (47%) |  |

1. Hunter S, Jones P, Mitchell A, Apweiler R, Attwood TK, et al. (2012) InterPro in 2011: new developments in the family and domain prediction database. Nucleic Acids Res 40: D306-312.
