# Supplementary material for: Involvement of MoVMA11, a Putative Vacuolar ATPase c’ Subunit, in Vacuolar Acidification and Infection-Related Morphogenesis of Magnaporthe oryzae
Source: PLoS One. 2013 Jun 27;8(6):e67804. doi: 10.1371/journal.pone.0067804 (PMC3694887; doi:10.1371/journal.pone.0067804)
Supplement: Table S3 — (DOC) [file pone.0067804.s009.doc]

**Table S3.** Effect of fermentable carbon sources on the diameter growth rate of the *ΔMovma11* mutant.

|  | **Sucrose** | **Fructose** | **Maltose** | **Xylose** | **Trehalose** |
| --- | --- | --- | --- | --- | --- |
| Wild-type | 101.3±4.4^A^ | 95.5±0.6^A^ | 100.0±1.6^A^ | 81.2±0.5^A^ | 98.8±2.3^A^ |
| *ΔMovma11* | 102.9±6.1^A^ | 95.9±2.7^A^ | 98.5±2.7^A^ | 84.2±5.1^A^ | 99.8±8.1^A^ |
| *Movma11c* | 94.7±4.4^A^ | 98.8±1.8^A^ | 99.9±1.0^A^ | 78.7±1.3^A^ | 95.6±2.3^A^ |

Diameter growth rate was determined as described in Table 1. The means followed by the same letter are not significantly different by Duncan’s multiple range tests at the 0.05 level of probability.
